# Etrasimod for alopecia areata: The scenario for a less extensive and moderate form

**DOI:** 10.1111/jdv.20693

**Published:** 2025-05-26

**Authors:** M. Starace

**Affiliations:** ^1^ Dermatology Unit IRCCS Azienda Ospedaliero‐Universitaria di Bologna Bologna Italy; ^2^ Department of Medical and Surgical Sciences Alma Mater Studiorum University of Bologna Bologna Italy

The scenario of treatment of alopecia areata (AA) has changed in the last years; thanks to the development of a new category of drugs named Janus Kinase inhibitors (JAKi) addressed in patients with severe forms of AA who are non‐responders to first‐line therapy.^1^ New molecules and drugs are approved in Phase 2/3 of clinical trials in patients with severe AA. Approved JAKi by the United States, European Union, Canada, China and Japan, with robust clinical trial data and peer‐reviewed publications, are baricitinib, a JAK1/2 inhibitor, in adult patients,^2^ and ritlecitinib, a selective dual inhibitor of JAK3 and all five members of the TEC family kinases, for adolescents (aged 12–17 years) and adult patients.^3^ Looking at this scenario of AA, there are still no approved indications for the less extensive and moderate form (Severity of Alopecia Tool [SALT] < 50) of the disease.

I read with great interest the article by King B and colleagues.^4^ The authors have conducted a Phase 2 randomized, double‐blind, placebo‐controlled trial for 52 weeks divided into a 24‐week double‐blind and a 28‐week open‐label extension period. The objectives were to evaluate the efficacy and safety of etrasimod in adult patients with moderate to severe AA. The drug studied is etrasimod, a daily oral, selective sphingosine 1‐phosphate 1, 4 and 5 receptor modulators. This drug has already been approved in patients with ulcerative colitis, but there are many ongoing studies on other inflammatory diseases including AA. The included patients were enrolled in two cohorts with different SALT values: In cohort 1, the randomization was 2:1 to etrasimod 2 mg or placebo of patients with SALT ≥50; in cohort 2, the randomization was 4:1:2 to etrasimod 3, 2 mg or placebo in patients with SALT ≥25 to <95.

The primary endpoint was to evaluate any modification of the percent of the SALT from baseline in each group with different dosages or placebo, and the secondary endpoint was an absolute change from the baseline of the SALT score at Week 24. In addition, the study considered the proportion of patients with ≥30% (SALT_30_), ≥50% (SALT_50_) and ≥75% (SALT_75_) improvement from baseline in the SALT score at Week 24. The treatment‐emergent adverse events (TEAEs) were mild and present in 74.7% with two cases of discontinuation.

The conclusions of this study were as follows^1^: the primary and secondary efficacy endpoints have not been achieved, showing no statistically significant results. At Week 24, the least squares mean showed the per cent of the SALT from baseline of the etrasimod 2 mg, 3 mg and placebo groups −13.8, −21.4 and 0.35, respectively. The least squares mean difference (95% CI; *p* value) in SALT score %CFB of etrasimod 2 and 3 mg versus placebo was −14.1 (−38.9 to 10.6; *p* = 0.2579) and − 21.8 (−44.4 to 0.9; *p* = 0.0592), respectively, and these results suggested a dose‐dependent effect^2^; efficacy was statistically relevant only versus placebo^3^; etrasimod was a safe and well‐tolerated drug for up to 52 weeks of treatment.

The study provides valuable insights into alopecia areata management; however, there are some critical points^1^: the duration of the current disease episode is a well‐recognized determinant of treatment response, as prolonged episodes are often associated with reduced therapeutic efficacy and increased resistance to intervention. A more detailed stratification based on episode duration could refine efficacy evaluation^2^; the use of a SALT score <20 as a treatment endpoint is clinically relevant, as it represents a threshold for meaningful hair regrowth and aligns with responder definitions in other clinical trials. Future studies should integrate these parameters to enhance therapeutic decision‐making and outcome prediction^3^; the study population reported a comparison with cohort 1, even if cohort 2 had milder AA, where a higher dosage was administered; and^4^ in conclusion, it seems that the absence of meeting the primary and secondary efficacy endpoints induced the authors to target this drug to moderate and milder cases of AA as a necessity for patients with less extensive (SALT score≤20) and moderate (SALT score 21 to 49) disease, based on the overall safety and tolerability emerging from the results obtained versus placebo.

## FUNDING INFORMATION

No funding supported the work.

## CONFLICT OF INTEREST STATEMENT

No conflict of interest.

## Data Availability

Data sharing not applicable to this article as no datasets were generated or analysed during the current study.
